# Effects of endurance exercise under hypoxia on acid-base and ion balance in healthy males

**DOI:** 10.20463/pan.2020.0015

**Published:** 2020-09-30

**Authors:** Sang-Seok Nam, Hun-Young Park

**Affiliations:** 1 Taekwondo Research Institute of Kukkiwon, Seoul, Korea; 2 Department of Sports Medicine and Science of Graduated School, Konkuk University, Seoul, Korea; 3 Physical Activity and Performance Institute (PAPI), Konkuk University, Seoul, Korea

**Keywords:** acute hypoxia, metabolic stress, capillary blood, potassium kinetics, submaximal exercise, blood lactate, blood glucose

## Abstract

**[Purpose]:**

This study was performed to investigate the acid-base and ion balance at rest and after exercise in healthy males under normoxia, moderate hypoxia, and severe hypoxia.

**[Methods]:**

Ten healthy Korean males completed three different trials on different days, comprising exercise under normoxia (F_i_O_2_ = 20.9%, N trial), moderate hypoxia (F_i_O_2_ = 16.5%, MH trial), and severe hypoxia (F_i_O_2_ = 12.8%, SH trial). They undertook endurance exercise for 30 min on a cycle ergometer at the same relative exercise intensity equivalent to 80% maximal heart rate under all conditions. Capillary blood samples were obtained to determine acid-base and ion balance at rest and after exercise.

**[Results]:**

Exercise-induced blood lactate elevations were significantly increased as hypoxic conditions became more severe; SH > MH > N trials (*P* = 0.003). After exercise, blood glucose levels were significantly higher in the SH trial than in the N and MH trials (*P* = 0.001). Capillary oxygen saturation (S_C_O_2_) levels were significantly lowered as hypoxic conditions became more severe; SH > MH > N trials (*P* < 0.001). The pH levels were significantly lower in the MH trial than that in the N trial (*P* = 0.010). Moreover, H_C_O_3_- levels were significantly lower in the SH trial than in the N trial, with significant interaction (*P* = 0.003). There were no significant differences in blood Na^+^, K^+^, and Ca^2+^ levels between the trials.

**[Conclusion]:**

MH and SH trials induced greater differences in glucose, lactate, S_C_O_2_, pH, and H_C_O_3_- levels in capillary blood compared to the N trial. Additionally, lactate, S_C_O_2_, and H_C_O_3_- levels showed greater changes in the SH trial than in the MH trial. However, there were no significant differences in Na^+^, K^+^, and Ca^2+^ levels in MH and SH trials compared to the N trial.

## INTRODUCTION

Exercise training in hypobaric hypoxia has been widely accepted as a useful method of enhancing the endurance performance of athletes, and a considerable amount of experimental evidence has accumulated regarding the efficacy of this training method [1-4]. Under hypobaric hypoxia, atmospheric pressure decreases; thus, the partial pressure of oxygen in the atmosphere decreases so that oxygen available in the same volume of air decreases, thereby inducing hypoxia [5,6]. Exercise under hypoxia induces lower arterial oxygen saturation and red blood cell deformability, whereas carbohydrate substrate mobilization and blood lactate levels during exercise are augmented [5,7-9]. In general, these physiological responses during exercise under hypoxia improve athletic performance and promote peripheral adaptation in the muscles [10]. However, the mechanism for these adaptive phenomena is not yet fully understood.

Hypoxia makes the energy supply more dependent on the glycolytic system during exercise by reducing the systemic oxygen supply, and these changes lead to metabolic acidosis through increased ATP synthesis by anaerobic metabolic processes, an increase of hydrogen ions, and a decrease in pH [11,12]. Exercise-induced accumulation of lactate and hydrogen ions reduces exercise performance [12,13]. Once hydrogen ions are produced in the muscles through exercise, they are buffered primarily by the bicarbonate (HCO_3-_) buffering system in the bloodstream [10]. Hypoxia rapidly increases minute ventilation and induces increased carbon dioxide removal, which relates to respiratory alkalosis [12,14]. Exercise in a hypoxic environment causes complex changes in the acid-base balance by HCO_3-_ and is simultaneously affected in terms of both metabolic acidosis induced by exercise and respiratory alkalosis induced by hypoxia [14]. Therefore, the exercise-induced kinetics of blood HCO_3-_ under hypoxia reflect the homeostasis of the acid-base balance in blood [10].

In addition to the acid-base balance, the ion balance is critical to muscle excitation, contraction, metabolism, and thus for muscle function during exercise [15]. The accumulation of extracellular potassium ions (K^+^) reduces the excitability of active muscles and lowers exercise tolerance [16,17]. Increased muscle Na^+^-K^+^ pump concentration via exercise training is usually associated with a reduced rise in plasma K^+^ during exercise [17]. Exercise under hypoxia is more dependent on anaerobic metabolism, with a concomitant increase in K^+^ during exercise in the blood, than exercise under normoxia [5,7]. These metabolic responses during exercise under hypoxia lead to acid-base responses, such as a concomitant release of K^+^ from the cell into extracellular fluid by increasing the opening of the muscular ATP-sensitive K^+^ channels [18,19]. In other words, exercise under hypoxia may augment exercise-induced alterations of acid-base and ion balances compared with exercise under normoxia. Lühker et al. [14] examined the differences in acid-base and ion balance during exercise under severe hypoxia (12% F_i_O_2_, equivalent to an altitude of 4500 m) and normoxia. They found a significant difference in pH and blood lactate levels during a 200 W cycle ergometer exercise between hypoxia and normoxia. However, Sumi et al. [10] compared the effects of acidbase and ion balance in response to high-intensity interval exercise under moderate hypoxia (14.5% F_i_O_2_, equivalent to an altitude of 3000 m) in endurance athletes. They reported that exercise under hypoxia did not elicit a decrease in blood pH or increase in K^+^ levels compared to an equivalent level of exercise under normoxia. The difference between these studies is the result of the difference in hypoxic conditions and exercise intensity.

Therefore, it is important to examine the differences in acid-base and ion balance during exercise under various hypoxic conditions. The purpose of this study was to evaluate the acid-base and ion balance at rest and after exercise in healthy males under normoxia, moderate hypoxia, and severe hypoxia. We hypothesized that exercise under moderate and severe hypoxia would induce greater exerciseinduced alterations of acid-base and ion balances compared with exercise under normoxia.

## METHODS

### Participants

Ten healthy Korean males participated in this study. The age, height, and body weight [mean ± standard deviation (SD)] of the participants were 29.6 ± 5.8 years, 176.2 ± 3.6 cm, and 62.9 ± 3.2 kg, respectively. The participants were nonsmokers and had no history of musculoskeletal, cardiovascular, or pulmonary diseases. In addition, they had not participated in any exercise program under hypobaric hypoxia or normobaric hypoxia in the previous six months. The participants provided their signed consent after sufficient explanation of the experiment and possible adverse effects. This study was approved by the Institutional Review Board of KyungHee University (KHSIRB 2015-020) in Korea. All procedures followed were in accordance with the ethical standards of the committee for responsible experimentation on humans and the Declaration of Helsinki.

### Study design

The participants visited the laboratory four times during the experimental period. During the first visit, all participants underwent fasting for more than 8 h, and after stabilization, height and body composition were measured in the morning. On the second, third, and fourth occasions, participants performed experimental trials in randomized order under normoxia (20.9% F_i_O_2_, equivalent to sea level, N trial), moderate hypoxia (16.5% F_i_O_2_, equivalent to an altitude of 2000 m, MH trial), and severe hypoxia (12.8% F_i_O_2_, equivalent to an altitude of 4000 m, SH trial). All participants completed endurance exercise for 30 min on a cycle ergometer (Aerobike 75XLII; Konami Corporation, Tokyo, Japan) at the same relative exercise intensity equivalent to 80% maximal heart rate (HRmax) using Miyashita formula (male = 209 - 0.69 × age) [20]. At rest and after exercise, capillary blood samples were obtained and analyzed. All experiments were performed in a 6.5 × 7.5 × 3 m environmental chamber (Submersible Systems, Huntington Beach, CA, USA) for all conditions. Various hypoxic conditions were simulated by introducing nitrogen into the environmental chamber using a nitrogen generator (Separation & Filter Energy Technology Cooperation, Siheung, Korea) with the capacity to simulate hypoxic conditions for altitudes of up to 9.7% O_2_, equivalent to an altitude of 6000 m. The temperature and humidity within the environmental chamber were maintained at 20 ± 2°C and 60% ± 2%, respectively, for all conditions. The three trials were spaced at least one week apart and performed at the same time of day, and the order of the trials was randomized.

### Measurements

*Body composition.* Body composition (i.e., height, body weight, fat-free mass, and % body fat) was measured after fasting for more than 8 h using bioelectrical impedance analysis equipment (Inbody 770, Inbody, Korea).

*Blood variables.* After 8 h of fasting, blood variables were measured at rest and immediately after exercise for 30 min. Blood glucose, lactate, capillary oxygen saturation (S_C_O_2_), pH, HCO_3-_, sodium (Na^+^), K^+^, and calcium (Ca^2+^) levels were measured using a blood gas analyzer (Gem Premier 3000, Radiometer, Denmark). For the acid-base and ion-balance measurements, blood samples were collected using the fingertip method in the capillary vessel. The acid-base and ion response results of the capillary blood were similar to those of the arterial blood, indicating the reliability of the results and that this is an easier sampling method compared to arterial blood sampling [21,22]. Additionally, blood samples were collected with the fingertip method after heating the capillary to 43°C, using an electric pad and massage for the influx of arterial blood, based on previous studies regarding heating the blood collection region and using stimulants, such as irritant cream, glyceryl trinitrate paste, and nicotinate paste [12]. After blood sampling, the responses of the acid-base and ion balance were evaluated within 10 s of closing the front and back of the collection tube to prevent the reaction of the blood sample with gases in the atmosphere.

### Statistical analysis

Means and standard deviations were calculated for all dependent variables. Normality of distribution of all outcome variables was verified using the Shapiro-Wilk test. Two-way analysis of variance (ANOVA) with repeated measures was used to assess the presence of interactions (trial × time) and main effects (trial or time). When ANOVA revealed a significant interaction or main effect, the least significant difference test was performed as a post hoc analysis to identify the differences. *P* <0.05 were considered statistically significant in all tests. All analyses were performed using the Statistical Package for the Social Sciences (SPSS) version 24.0.

## RESULTS

### Blood variables

#### Metabolites

**Fig. 1.**
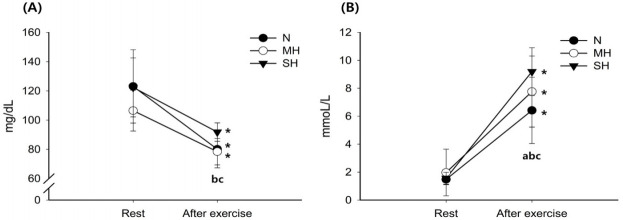
Changes in blood glucose (A) and lactate (B) concentrations. Values are mean ± standard deviation (SD). ^*^significant difference compared with pre-exercise. ^a^Significant difference between normoxia (N) and moderate hypoxia (MH) trials. ^b^Significant difference between N and severe hypoxia (SH) trials. ^c^Significant difference between MH and SH trials.

Figure 1 shows the changes in blood glucose and lactate levels. Blood glucose levels decreased significantly with exercise in all trials (main effect for time, *P* < 0.001). After exercise, blood glucose levels were significantly higher in the SH trial than in the N and MH trials (main effect for trial, *P* = 0.001). The blood lactate levels increased significantly with exercise in all trials (main effect for time, *P* < 0.001). Exercise-induced blood lactate level elevations were significantly increased as the hypoxic conditions became more severe; SH > MH > N trials (interaction, *P* = 0.001; main effect for trial, *P* = 0.003).

#### Blood S_C_O_2_, pH, and HCO_3-_

**Fig. 2.**
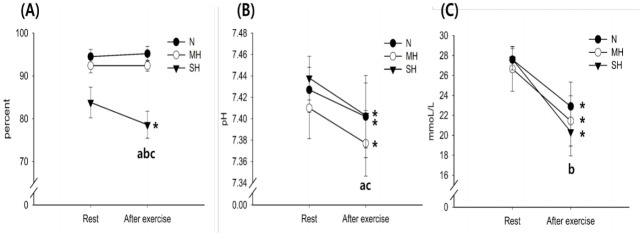
Changes in S_C_O_2_ (A), pH (B), and HCO_3-_ (C) levels. Values are mean ± standard deviation (SD). ^*^Significant difference compared with pre-exercise. ^a^Significant difference between normoxia (N) and moderate hypoxia (MH) trials. ^b^Significant difference between N and severe hypoxia (SH) trials. ^c^Significant difference between MH and SH trials.

Figure 2 illustrates the changes in blood S_C_O_2_, pH, and HCO_3-_ levels. The S_C_O_2_ levels decreased significantly with exercise in the SH trials (main effect for time, *P* = 0.027). After exercise, S_C_O_2_ levels were significantly reduced as the hypoxic condition became more severe; SH > MH > N trials (main effect for trial, *P* < 0.001). The pH levels decreased significantly with exercise in all trials (main effect for time, *P* = 0.010). After exercise, pH levels were significantly lower in the MH trial than in the N trial (main effect for trial, *P* = 0.010). The HCO_3-_ levels decreased significantly with exercise in all trials (main effect for time, *P* < 0.001), and there was a significant interaction between time and trial (*P* = 0.003).

#### Blood Na^+^, K^+^, and Ca^2+^

**Fig. 3.**
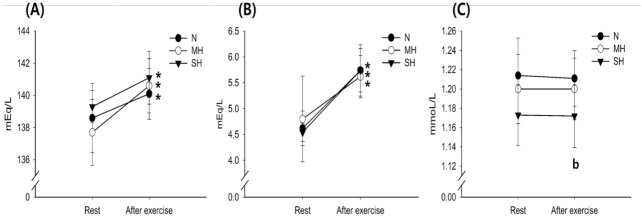
Changes in Na^+^ (A), K^+^ (B), and Ca^2+^ (C) concentrations. Values are mean ± standard deviation (SD). ^*^Significant difference compared with pre-exercise. ^a^Significant difference between normoxia (N) and moderate hypoxia (MH) trials. ^b^Significant difference between N and severe hypoxia (SH) trials. ^c^Significant difference between MH and SH trials.

Changes in blood Na^+^, K^+^, and Ca^2+^ levels with exercise for all trials are shown in Figure 3. The Na^+^ and K^+^ levels increased significantly after exercise in all trials (main effect for time, *P* < 0.001), whereas there were no significant differences between trials at any time point. The Ca^2+^ levels were significantly lower in the SH trial than in the N trial (main effect for trial, *P* = 0.019).

## DISCUSSION

According to our hypothesis, exercise under moderate and severe hypoxia would induce greater differences in glucose, lactate, S_C_O_2_, pH, and HCO_3-_ levels in capillary blood compared with exercise under normoxia. In addition, lactate, S_C_O_2_, and HCO_3-_ levels showed greater changes in the SH trial than the MH trial. However, there were no great differences in Na^+^, K^+^, and Ca^2+^ levels in MH and SH trials compared with the N trial.

Hypoxia decreases the systemic oxygen-carrying capacity and peripheral oxygen-utilizing capacity, making it more dependent on glycolysis; these changes induce an increase in blood glucose levels, blood lactate levels, and respiratory exchange ratio during exercise [8,23]. Exposure to and exercise under hypoxia induce a decrease in oxygen saturation in the arterial blood, resulting in an augmented energy supply from the glycolytic system, increasing the blood lactate level and production of hydrogen ions (H+) [9,10,24]. The augmented metabolic stress stimulates the opening of ATP-sensitive K^+^ channels in the muscle, subsequently increasing K^+^ outflow to the extracellular fluid in the active muscle [24,25]. Additionally, proton production is further increased during exercise under hypoxia; protons are then released into the bloodstream, and the blood is buffered by the HCO_3-_ buffer system [14,15]. Moreover, the acid-base response by the HCO_3-_ buffer system is caused by an increase in ventilation during exercise [12]. Lühker et al. [14] investigated the differences in acid-base and ion balance during exercise under severe hypoxia (12% F_i_O_2_, equivalent to an altitude of 4500 m) and normoxia, and hypoxia showed a significant decrease in pH and increase in blood lactate levels during a 200 W cycle ergometer exercise compared to normoxia. In our study, SH showed a greater blood glucose level after exercise compared to the MH and N trials, and exercise-induced blood lactate level elevations were significantly increased as the hypoxic conditions became more severe; SH > MH > N trials. In addition, the MH and SH trials showed a lower S_C_O_2_, pH, and HCO_3-_ than the N trial after exercise. These findings were consistent with those of Sumi et al. [10] and Woorons et al [26].

Regarding the ion balance, our initial hypothesis was that the MH and SH trials would augment the exercise-induced blood K^+^ elevation compared with the N trial. Because lower S_C_O_2_ and pH increases the opening of K_ATP_ channels, we believed that K^+^ would efflux from the working muscle into the bloodstream [24,27]. The increased accumulation of extracellular K^+^ during exercise under hypoxia is manifested by the enhanced release of K^+^ in the working muscle and decreased reuptake of K^+^ during exercise [24]. Street et al. [18] verified that extracellular K^+^ accumulation and metabolic alkalosis were strongly correlated; their findings seem reasonable, considering the significant increase in K^+^ and Ca^2+^ induced by exercise in all trials (SH, MH, and N) in our study.

However, our results did not show a significant difference in K^+^ after exercise between SH and MH trials and the N trial. Although the exercise type and intensity differed, results reported by Sumi et al. [10] and Sumi et al. [24] regarding ion balance were similar to those of our study. Sumi et al. [10] investigated the effects of acid-base and ion balance in response to high-intensity interval exercise under moderate hypoxia (14.5% F_i_O_2_) and normoxia in endurance athletes. They reported that exercise under hypoxia did not elicit a decrease in blood pH and increase in K^+^ levels compared with an equivalent level of exercise under normoxia. The difference between these previous studies is due to the difference in hypoxic conditions and exercise intensity. Additionally, Sumi et al. [24] investigated the acid-base balance and K^+^ kinetics in response to exercise (relative exercise intensity) under moderate hypoxia (14.5% F_i_O_2_) among endurance athletes (age: 20.7 ± 0.9 years, height: 172.5 ± 2.2 cm, body mass: 61.6 ± 2.8 kg). They reported that endurance exercise under moderate hypoxia elicited a decline in venous pH; however, it did not induce an increase in blood K^+^ levels compared with normoxia. These two previous studies suggest that exercise (high-intensity interval exercise and endurance exercise) under moderate hypoxia cause greater metabolic stress and similar exercise-induced elevation of blood K^+^ compared with the same exercise under normoxia, despite lower exercise absolute load. These findings were confirmed in our study examining the effects of exercise under moderate and severe hypoxia on the acid-base and ion balance in healthy males compared with exercise under normoxia. In other words, our study confirmed that severe hypoxic conditions induced a greater metabolic response during exercise, without affecting the ion balance, such as K^+^, Na^+^, and Ca^2+^ kinetics, during exercise compared to normoxia.

In conclusion, the MH and SH trials induced greater differences in glucose, lactate, S_C_O_2_, pH, and HCO_3-_ levels in capillary blood compared to the N trial. Additionally, lactate, S_C_O_2_, and HCO_3-_ levels showed greater changes in the SH than in the MH trial. However, there were no significant differences in Na^+^, K^+^, and Ca^2+^ levels in MH and SH trials compared with the N trial.
